# BALANCING HEALTH AND CAREGIVING: ADULT CHILDREN’S HEALTH PROBLEMS IMPACT ON CAREGIVING

**DOI:** 10.1093/geroni/igae098.1293

**Published:** 2024-12-31

**Authors:** Catherine Stepniak, J Jill Suitor, Megan Gilligan

**Affiliations:** University of Lynchburg, Lynchburg, Virginia, United States; Purdue University, West Lafayette, Indiana, United States; University of Missouri, Columbia, Missouri, United States

## Abstract

When older mothers face chronic or acute health problems their adult children often become caregivers, serving as either as the primary or secondary sources of informal care. However, when adult children are facing their own serious health problems, their ability to serve as caregivers may be limited. Surprisingly, the literature provides no clear insight into whether children’s health affects their provision of care to their mothers. In the present study, we shed new light on this question using quantitative and qualitative data collected from 412 adult children nested within 224 families as part of the Within-Family Differences Study. Quantitative analyses showed that adult children with health problems were as likely as their healthy siblings to serve as their mothers’ primary and secondary caregivers. Consistent with the literature, daughters were substantially more likely than sons to serve as caregivers. Qualitative analyses revealed that this gendered pattern occurred even in families in which daughters had serious health problems and sons were healthy. Daughters’ explanations for becoming caregivers despite their health limitations indicated that they had internalized messaging that they were the “naturally better” caregivers compared to their brothers. Adult children caregivers reported higher level of caregiver burden than that reported by healthy caregivers as they shouldered joint responsibility for their own and their mothers’ care. These findings underscore the ways in which gendered expectations for care and health problems across generations intersect to increase the risk of caregiver burden and unmet care needs for members of both generations.

